# Determinants of health facility choice for delivery among women participating in group antenatal care in Machakos county, kenya: A cross-sectional survey

**DOI:** 10.1186/s12978-025-02107-w

**Published:** 2025-08-22

**Authors:** Jefferson Mwaisaka, Pooja Sripad, Melanie Olum, Patricia Owira, Samuel Mwaura, Rhoda Njeru, Daniel Muli, Faith Mutisya, Anne Hyre, Lisa Noguchi, Stephanie Suhowatsky

**Affiliations:** 1 International Center for Reproductive Health, Mombasa, Kenya; 2 Jhpiego, DC, WA USA; 3 Jhpiego, Nairobi, Kenya; 4 County Health Management, Machakos, Kenya

**Keywords:** Group antenatal care, Delivery choice, Kenya

## Abstract

**Background:**

Group antenatal (G-ANC) care has been introduced in Kenya and is associated with increased ANC contacts. Previous studies did not report higher likelihood of facility-based deliveries, where facility delivery rates are high (> 80%). It is unknown whether G-ANC influences women’s choice of delivery facility. This study sought to understand if and how exposure to and experience of G-ANC, among other factors, influence women’s decisions on which facility to deliver. Study findings can inform health system strategies including the Service Delivery Re-design.

**Methods:**

We conducted a cross-sectional survey from December 2023 to January 2024 in eight health centers in Machakos County, Kenya with women who participated in G-ANC and had a facility delivery to understand women’s choice of health facility for childbirth, i.e., whether they returned to the same G-ANC facility for delivery or went to another facility. The survey interviews were administered through telephone, as a nested component of a larger implementation research focused on the adoption of G-ANC.

**Results:**

Of the 470 women who participated in the phone survey, 29.8% of women returned to deliver at the facility where they received G-ANC and 70.2% delivered elsewhere. Most women (84.3%) delivered in the public sector. Regression analysis models revealed two significant predictors for delivery in the same G-ANC facility: proximity to the facility (adjusted AOR 4.13, 95% CI: 2.73–6.23, *p* < 0.001); and positive staff attitudes (adjusted AOR 4.68, 95% CI: 2.06–10.60, *p* < 0.001). Two significant predictors of delivering in a different facility were being “told/advised to give birth there during pregnancy/ANC” (adjusted AOR 0.23, 95% CI: 0.15–0.35, *p* < 0.001) and high household wealth status (quintile four AOR 0.29, 95% CI: 0.09–0.91, *p* = 0.034; quintile five AOR 0.23, 95% CI: 0.07–0.72, *p* = 0.011). Intervention exposure was not significant.

**Conclusion:**

Most women participating in G-ANC chose to deliver in another facility. The choice of facility for childbirth was most strongly influenced by factors other than the intervention, such as proximity, positive staff attitudes, health advice, and household wealth status.

**Supplementary Information:**

The online version contains supplementary material available at 10.1186/s12978-025-02107-w.

## Abstract (plain language summary)

Group antenatal care (G-ANC) is an alternative service delivery model introduced in select public health facilities in Kenya. G-ANC was not associated with increased facility delivery in Kenya, where most women (over 80%) already deliver in health facilities. However, it’s unclear if and how G-ANC affects women’s choice of where to give birth, which is important for understanding care-seeking behaviors for maternal services as health systems invest in G-ANC and other health systems strengthening strategies. We surveyed 470 women in Machakos County who participated in G-ANC in eight study health centers and delivered in a health facility. The goal was to find out where they delivered and whether G-ANC influenced that decision. The survey was conducted by phone between December 2023 and January 2024 as part of a larger study on G-ANC. The results showed that 70.2% delivered at a different facility and 29.8% delivered at their G-ANC facility. Women were more likely to choose a different facility if they were advised to deliver there during pregnancy or if they came from wealthier households. Women were more likely to deliver at their G-ANC facility if it was close to home or if they had positive experiences with staff there. The G-ANC intervention did not directly influence facility choice. Findings suggest that proximity, staff attitudes, health advice, and household wealth influence delivery facility choice more than other factors. These findings can guide future county and national programming on G-ANC and other health system strategies aiming to improve maternal health outcomes.

**Supplementary Information:**

The online version contains supplementary material available at 10.1186/s12978-025-02107-w.

## Background

The access to quality antenatal care (ANC) is an important step towards improving maternal and perinatal health outcomes, particularly in low-and middle-income countries (LMICs) such as Kenya, where facility delivery rates are at 88% but maternal and neonatal mortality remain significant challenges [11] [15, 16]. Regular adherence to ANC services can potentially detect complications early, promote healthy behaviors, and ensure timely interventions, which can significantly improve birth outcomes. A higher frequency of ANC contacts has been associated with increased likelihood of delivering in a health facility and other positive intermediate outcomes including skilled birth attendance and early detection of complications [9]. A 2009 framework for increased use of delivery care services identified ANC use and other factors (e.g., previous facility birth, higher maternal age, higher education, greater household wealth, lower parity, urban residence) [6] Obstetric complications, quality of care, and distance to the health facility as likely influences.

Group Antenatal Care (G-ANC) is an alternative service delivery model that enhances the experience and provision of care and is associated with increased numbers of ANC contacts [7, 8]. G-ANC brings together pregnant women in small groups for antenatal clinical consultations, group education, and social support. Conventional ANC involves individual consultations between a pregnant woman and a healthcare provider. G-ANC takes a different approach by organizing women into small groups based on their gestational age and scheduling subsequent ANC contacts as a group. In G-ANC, women receive individual private clinical consultations from a healthcare provider, as well as interactive facilitated group discussions which builds peer support and shared health promotion. This model fosters peer learning, emotional support, and a participatory approach to health education [7]. Studies in sub-Saharan Africa in Kenya [7, 8, 24], Nigeria [7, 8], Senegal [17], and Ghana [13, 14] have shown that G-ANC can improve health literacy, satisfaction, ANC attendance, and health outcomes by offering a more holistic and supportive approach to care. A trial in Nigeria and Kenya reported an association between G-ANC and increased facility deliveries, but this effect was observed only in Nigeria, where pre-trial facility delivery rates were low. [8].

Where G-ANC is provided and facility delivery is prevalent, there is limited understanding of the factors that influence which facility they choose for childbirth. It is possible that G-ANC may influence women to return to the same G-ANC facility for delivery because: women have a positive experience in G-ANC; they feel cared for, supported and respected at the G-ANC facility; and they form supportive relationships with the ANC healthcare providers, who often also provide intrapartum care in health centers in the Kenyan context. [7] It is also possible that G-ANC influences women to choose another facility by: increasing health literacy around recognizing danger signs and actions for birth preparedness and complications readiness (e.g., choosing a facility to give birth and for seeking care in case of complications); and building women’s confidence to take actions, including care-seeking decisions for childbirth (e.g., going to a hospital in case of pre-term birth).

In Kenya where most women give birth in a health facility, G-ANC is one of several health systems interventions to improve use of quality Maternal and Newborn Health (MNH) services and reduce mortality, including: Service Delivery Redesign (SDR) to ensure women give birth close to Comprehensive Emergency Obstetric and Newborn Care (CEmONC); maternal health insurance programs as part of Universal Health Coverage; and Continuum of Care - which emphasizes integrated, timely, and coordinated maternal and newborn health services from pregnancy through childbirth and the postnatal period [20]. This study seeks to explore if and how G-ANC shapes women’s facility choices for childbirth. This information would be helpful to policymakers so they can design various health system strategies to be synergistic to improve maternal, perinatal and neonatal outcomes.

This study was a nested sub-study within an implementation research aimed at describing the feasibility, acceptability, and effectiveness of adopting G-ANC and integrating into routine county systems as the predominant model of care (Mwaisaka, Owira, Olum, et al., 2024, in press) in Machakos county, Kenya. This paper reports on G-ANC participants’ choice of health facilities for delivery. It answers three research questions: (1) Do G-ANC participants deliver in the same facilities where they attended G-ANC or another facility (Yes/No)? (2) What factors influence where G-ANC participants give birth? (3) Is greater intervention exposure associated with birth in the same facility where G-ANC was provided to the client (i.e., dose-response relationship)?

## Methods

### Study design and setting

We conducted a cross-sectional survey among women who participated in G-ANC and had delivered in a facility to explore if women returned to their G-ANC facility for delivery or went to another facility The survey was nested within a broader implementation research effort on assessing the adoption of G-ANC in Machakos County (Mwaisaka, Owira, Olum, et al., 2024, in press). It was conducted in all eight study health centers providing G-ANC and offer basic emergency obstetric and newborn care (BEmONC) services, with an average monthly caseload of 54 ANC clients and 20 deliveries per month between March 2022 and November 2023, based on data extracted from facility registers. Four hospitals in the main study were excluded from the sub-study because they provided comprehensive emergency obstetric and newborn care (CEmONC) services, which would likely bias women’s decisions.

Machakos county, adjacent to the national capital Nairobi, has a population of 1.4 million people who are primarily working in agriculture, forestry and fishing [27]. ANC and intrapartum care are provided mainly in the 61 Level three, 30 Level four, and one Level five Hospital in Machakos [28]. Level four hospitals and the Level five county hospital provide ANC with specialized care for clients with complications, have laboratory and ultrasound capacity, and provide CEmONC. Health centers (i.e., Level three and four facilities in the Kenyan health system) provide ANC and BEmONC. All study facilities were accredited by the National Health Insurance Fund, now the Social Health Insurance Fund and provide ANC and delivery care for free to registered women [30].

### Participant sample

Study participants consisted of postpartum women enrolled in G-ANC during pregnancy, attended at least one G-ANC meeting, and had an estimated delivery date within the past five months, calculated from the start of data collection. We allocated the number of women to be surveyed at each facility in proportion to the number of G-ANC participants recorded at that facility in the main study. To meet the sample size, we estimated that women enrolled in G-ANC over a five- to six-month period would need to be screened for attendance eligibility.

Sample size calculation was based on the estimated proportion of G-ANC participants that gave birth in same facility where they were exposed to G-ANC. In the absence of empirical data, we used the conservative estimate and approximation by healthcare providers working at the eight participating health centers of 50% (i.e., 10 out of 20 women per G-ANC cohort) returning to their G-ANC facility for birth - as a pragmatic proxy for the calculation. It is important to note that this estimate did not rely on surveying women who had already delivered, but rather reflected provider insights into typical delivery patterns. The nested nature of this sub-study restricted our sampling frame to G-ANC participants from these sites. This approach limits generalizability to the wider population, but it aligns with the study’s objective of exploring delivery facility decision-making among women exposed to G-ANC. An inter-class coefficient (ICC) of 0.03 was estimated using individual data from prior surveys conducted with the G-ANC participants [8] and accounting for cluster-specific features such as proximity to a health facility that could influence the proportion of deliveries in the same facilities. A design effect of 2 was calculated based on ICC of 0.03 and 10 women per cohort per facility likely to deliver in the same G-ANC facilities. The sample size of 392 women was calculated with a 7% margin of error (assuming a simple random sample) and adjusted for 20% non-response rate, for a final study sample size of 470 women.

### Selection and recruitment

Participants were identified from eligible G-ANC cohorts, using the intervention register (i.e., G-ANC cohort tracker). Women’s estimated delivery dates were used to select cohorts that delivered within five months preceding data collection. Inclusion criteria included: participation in at least one G-ANC meeting; delivery in a health facility within the five months prior to data collection; 18 years of age or older; in Machakos County at the time of labor; able and willing to provide informed consent; and able to speak English or Swahili. Women who had no phone numbers written in the intervention register and with no mental capacity to provide consent were excluded from the study.

From August 2023, G-ANC facilitators, often a nurse working in the facility, read a brief description of the study during routine G-ANC meetings to inform women attending the G-ANC meetings in the eight health centers that they may be contacted by phone after they had given birth and requested to participate in this study. During data collection, research assistants identified eligible G-ANC cohorts that had estimated delivery dates within the past five months using the intervention register. We contacted eligible participants using the phone contacts they provided in the intervention register (cohort tracker). Women who were identified and reached were screened for eligibility and their interest to participate. They were verbally consented to participate prior to phone survey administration.

### Data collection

We conducted data collection between December 2023 and January 2024. We engaged research assistants experienced in quantitative field research for this exercise. The telephone administered survey questionnaire was designed by the International Centre for Reproductive Health - Kenya (ICRHK) and Jhpiego teams modified from a similar study being implemented in Nigeria (Johns Hopkins School of Public Health Institutional Review Board #22639). It was translated into Swahili (most popular language in Kenya) and asked about basic demographic information, where they delivered, the reasons or factors that influenced this choice, and exposure to G-ANC (i.e., the number of meetings attended). Questions about the factors were first open-ended to capture different reasons. The survey was administered through telephone interviews and data was captured electronically using SurveyCTO platform [29]

### Data analysis

A research statistician checked the data for completeness and consistency prior to data analysis. Background characteristics of study participants are presented using descriptive statistics. Categorical variables were checked for differences between the two groups (i.e., delivery in the same G-ANC facility or in a different facility) using chi-square tests. The primary outcome assessed was a binary variable indicating whether women returned to their G-ANC facility for delivery or not. During analysis, the outcome was coded as “1” for delivering at the same facility and “0” for delivering at a different facility. Given the exploratory nature of the study, several independent variables of interest were drawn from the all the factors that participants provided as reasons for their choice of facility. Factors were categorized by: health status related to labour complications; access to healthcare services; facility experience; social/cultural factors; and determinants associated with the G-ANC intervention. Study facilities were classified by size of their recruited ANC participants and ordered from low to high caseloads. We adjusted for socio-demographic covariates (age, education, marital status, wealth status) identified a priori as potential confounders and assessed access-related indicators (transport, cost, proximity), and facility experience (staff attitudes, trust, good experience/being treated respectfully), and liking one’s G-ANC experience.

Bivariate chi-squared tests were employed to initially explore associations between these independent variables and the outcome of interest. Variables showing non-significant associations (p-values > 0.05) or extreme counts in response categories (< 5% or > 95%) were excluded to ensure data variability and model robustness. Redundant variables or those exhibiting strong inter-item correlation were also excluded. The final logistic regression model was iteratively built using the Generalized Estimating Equation in STATA version 15 [30], to account for intra-facility correlation and potential clustering of data at the facility level. Multivariable logistic regression with a 95% confidence interval was used to assess the relationships between independent variables and the study’s outcome of interest. Findings are presented as odds ratios. Results were statistically significant at *p* < 0.05. All quantitative data analysis was done using STATA version 15 [26].

### Ethical approval

This study was conducted in accordance with the ethical principles outlined in the Declaration of Helsinki. It received ethical review and approval from Johns Hopkins Bloomberg School of Public Health Institutional Review Board, Baltimore, Maryland, USA (21094) and AMREF Ethics and Scientific Review Committee, Nairobi, Kenya (P1219-2022). Research assistants received a four-day training on ethical conduct of research including the purpose, risks, benefits, and voluntary nature of the study, as well as the informed consent process.

## Results

A total of 470 women participated in the phone survey. Women were an average of 26 years (SD = 5 years), mostly completed secondary school (60%), and about half were employed (52%) in the past 12 months. (Table 1) Approximately one-quarter of the women were from lower-wealth quintile households, while over half were in the highest two quintiles (e.g., quintile 4 at 28.9%, quintile 5 at 23.8%). Most (83%) were married and already mothers: one child (39%), two children (34%) and three or more children (28%). Just over two-thirds of the surveyed women (68%) had been pregnant once or twice.

**Fig. 1:**
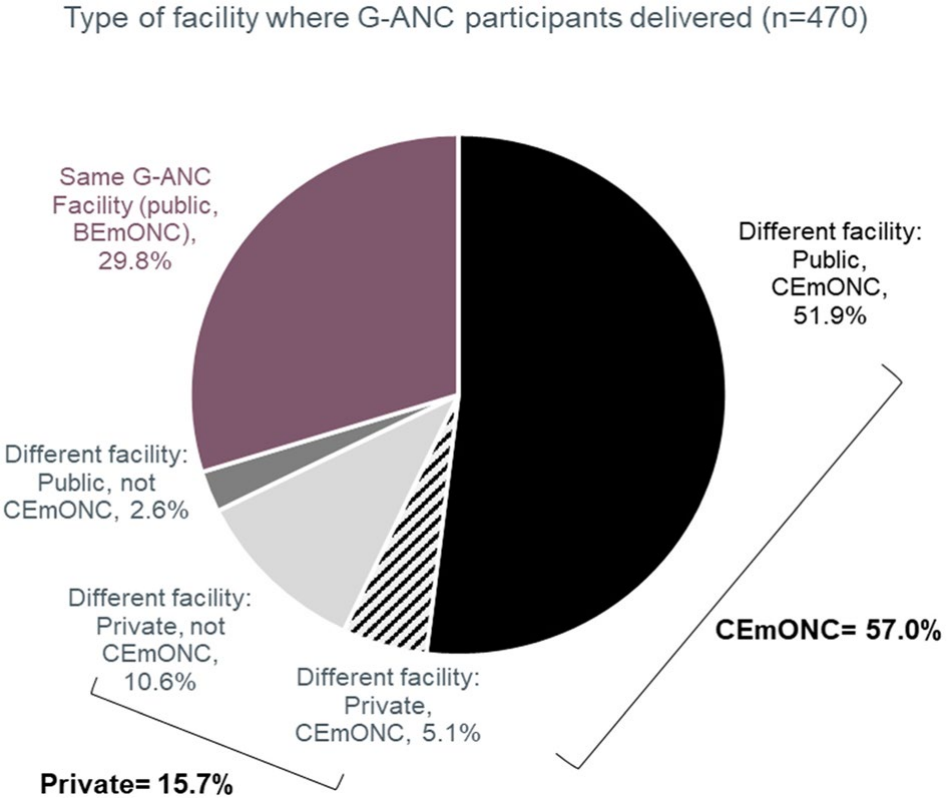
Type of facility where G-ANC participants delivered

Overall, 29.8% of women returned to deliver at the facility where they received G-ANC, and 70.2% delivered elsewhere (Fig. 1). Most women (84.3%) delivered in the public sector compared to 15.7% in the private sector. Over half (57.0%) of women gave birth in a CEmONC facility. Among those who delivered in a different facility, the majority (81.2%) gave birth in CEmONC facilities, mainly public facilities (Fig. 1).

Chi-squared tests revealed a few socio-demographic differences between women returning to same G-ANC facilities and those going to different facilities. Most women returning to the same facility were in the 20–24 years (34%) and 25–30 years (30%) groups and had a secondary education level (54%). Additionally, women in the lowest two quintiles were more likely to give birth in the same facility, while women who gave birth in a different facility were more likely to be in the two highest quintiles. Also, facilities with higher ANC caseloads were associated with increased proportions of women giving birth in a different facility. The level of exposure to G-ANC (i.e., number of meetings attended) was not a significant factor in facility choice (Table 1).

Women were asked if G-ANC influenced where they gave birth, and 59% of all respondents said it did. A significantly higher proportion of those who gave birth in the same facility where they attended G-ANC reported this influence (90%), compared to 45% of those who gave birth in another facility (*p* < 0.001).

**Table 1:** Characteristics of women surveyed postpartum who attended G-ANC in eight health centers in machakos, Kenya (*n* = 470)

Characteristics		Delivered at same (G-ANC) facility (*n* = 140) Number (%)	Delivered at a different facility (*n* = 330) Number (%)
Age	19 and below	8 (5.7)	24 (7.3)
	20–24 years	47 (33.6)	105 (31.8)
	25–30 years	42 (30.0)	129 (39.1)
	31 years and above	30 (21.4)	63 (19.1)
	Missing age	13 (9.3)	9 (2.7)
Highest education level	Primary and below	43 (30.7)	61 (18.5)
	Secondary	75 (53.6)	208 (63.0)
	College & university	22 (15.7)	61 (18.5)
Employed (ref = not employed)		73 (52.1)	169 (51.2)
Married (ref = not married)		114 (81.4)	276 (83.6)
Wealth quintiles	Quintile 1	31 (22.1)	34 (10.3)
	Quintile 2	27 (19.3)	30 (9.1)
	Quintile 3	30 (21.4)	70 (21.2)
	Quintile 4	33 (23.6)	103 (31.2)
	Quintile 5	19 (13.6)	93 (28.2)
Live children	One child	45 (32.1)	136 (41.2)
	Two children	49 (35.0)	109 (33.0)
	Three or more	46 (32.9)	85 (25.8)
Number of pregnancies	One	42 (30.0)	119 (36.1)
	Two	48 (34.3)	110 (33.3)
	Three	30 (21.4)	63 (19.1)
	Four and more	20 (14.3)	38 (11.5)
G-ANC Facility (increasing caseload)	Facility 1	8 (5.7)	20 (6.1)
	Facility 2	4 (2.9)	25 (7.6)
	Facility 3	17 (12.9)	16 (4.9)
	Facility 4	24 (17.1)	10 (3.0)
	Facility 5	18 (12.9)	18 (5.5)
	Facility 6	25 (17.9)	49 (14.9)
	Facility 7	19 (13.6)	60 (18.2)
	Facility 8	25 (17.9)	132 (40.0)
Number of G-ANC meetings attended	1–2 meetings	32 (22.9)	78 (23.6)
	3–4 meetings	54 (38.6)	126 (38.2)
	5–7 meetings	54 (38.6)	126 (38.2)

## Comparative distributions of factors that influenced the choice of delivery facilities

Women were asked to describe all the factors that influenced their choice of facility for delivery. Supplemental Table 1 provides a detailed distribution of all factor distributions (multiple responses) analyzed in relation to the choice of delivering at the same G-ANC or a different facility. Statistically significantly different distributions of factors influencing facility choice are presented in Table 2.

The chi-square analysis of factors reveals that three factors are significantly associated with higher likelihood of delivering at the same G-ANC facility: proximity to residence (*p* < 0.001); affordability (*p* = 0.005); and a positive experience (staff attitudes, trust, respectful treatment) with G-ANC (*p* < 0.001). Two factors are significantly associated with higher likelihood of delivering in a different facility: advice received during pregnancy (*p* = 0.004); and personal preferences (*p* = 0.017). (Table 2)

**Table 2:** Distribution of factors influencing delivery facility choice among women attending G-ANC in machakos, Kenya (*n* = 470)

Factor/variable influencing choice at most recent delivery		Delivered at the same (G-ANC) facility (*n* = 140) Number (%)	Delivered at a different facility (*n* = 330) Number (%)
Health	Was told/advised to give birth there during pregnancy/ANC	10 (7.1)	57 (17.3)^**^
Access	A place I could afford to give birth (cost)	20 (14.3)	21 (6.4)**
	Based on available transport	21 (15.0)	29 (8.8)*
	Closest facility to my residence	62 (44.3)	51 (15.6)***
Facility experience	Staff attitudes	37 (26.4)	20 (6.1)***
	Trust	21 (15.0)	18 (5.5)**
	Good experience here (treated respectfully)	43 (30.7)	32 (9.7)***
G-ANC Intervention	Liked G-ANC experience at this facility	63 (45.0)	38 (11.5)***
Other (social/cultural)	Personal preference	10 (7.1)	50 (15.2)*

Key: *p < 0.05; ** p < 0.01; *** p < 0.001

## Bivariate and multivariate associations with returning to the same G-ANC facility for delivery

Bivariate and multi-level multivariate logistic regression models used to assess the relative influences on a woman’s choice to return to the same G-ANC facility for delivery revealed two significant predictors. Proximity to the facility strongly influenced the decision, with a high likelihood of returning to the same facility (unadjusted OR 3.91, 95% CI: 2.53–6.04, *p* < 0.001; adjusted AOR 4.13, 95% CI: 2.73–6.23, *p* < 0.001). Positive staff attitudes were also significantly associated with returning to the same facility in both models (unadjusted OR 5.07, 95% CI: 1.75–14.66, *p* = 0.003; adjusted AOR 4.68, 95% CI: 2.06–10.60, *p* < 0.001). Positive facility experiences (treated respectfully) were significant in the bivariate analysis, but did not remain significant in the adjusted model. (Table 3)

Two factors were significant predictors of delivering in a different facility (i.e., not the same G-ANC facility): was being told/advised to give birth there during pregnancy/ANC (unadjusted OR 0.42, 95% CI: 0.28–0.63, *p* < 0.001; adjusted AOR 0.23, 95% CI: 0.15–0.35, *p* < 0.001) and higher household wealth status for women in the two highest quintiles (quintile four AOR 0.29, 95% CI: 0.09–0.91, *p* = 0.034; quintile five AOR 0.23, 95% CI: 0.07–0.72, *p* = 0.011) (Table 3).

**Table 3:** Bivariate and multivariate associations with returning to the same G-ANC facility for delivery (ref = other facility) (*n* = 470)

Variable	Delivered at the same (G-ANC) facility Unadjusted Odds Ratio (95% CI)	Delivered in the same (G-ANC) facility Adjusted Odds Ratio (95% CI)
Health: told/advised to give birth there during pregnancy/ANC	0.42 (0.28, 0.63)***	0.23 (0.15, 0.35)***
Access: place I could afford to give birth (cost)	1.93 (0.88, 4.25)	1.07 (0.38, 3.01)
Access: Based on available transport	1.65 (0.87, 3.14)	0.97 (0.42, 2.23)
Access: Closest facility to my residence	3.91 (2.53, 6.04)***	4.13 (2.73, 6.23)***
Facility experience: Staff attitudes	5.07 (1.75, 14.66)**	4.68 (2.06, 10.60)***
Facility experience: Trust	2.19 (0.82, 5.87)	0.56 (0.12, 2.69)
Facility experience: Good experience here (treated respectfully)	4.45 (1.55, 12.80)**	3.54 (0.88, 14.25)
G-ANC intervention: Liked G-ANC experience at this facility	3.51 (1.53, 8.02)**	2.22 (0.74, 6.65)
Other: Personal preference	0.72 (0.22, 2.42)	0.48 (0.15,1.56)
Age		
Ref: 19 and below		
20–24 years	0.90 (0.35, 2.32)	0.80 (0.15, 4.31)
25–30 years	0.95 (0.45, 1.99)	0.82 (0.19, 3.54)
31–46 years	0.83 (0.32, 2.17)	1.07 (0.18, 6.40)
Highest education level		
Ref: Primary and below		
Secondary	0.66 (0.44, 0.98)	0.93 (0.44, 1.98)
College and university	0.67 (0.44, 1.02)	1.24 (0.58, 2.68)
Wealth quintile		
Ref: Quintile 1		
Quintile 2	1.31 (0.69, 2.48)	0.90 (0.41, 2.00)
Quintile 3	0.92 (0.68, 1.23)	0.48 (0.19, 1.25)
Quintile 4	0.74 (0.37, 1.47)	0.29 (0.09, 0.91)*
Quintile 5	0.51 (0.33, 0.79)**	0.23 (0.07, 0.72)*

Key: *p < 0.05; ** p < 0.01; *** p < 0.001

## Discussion

We examined delivery facility choices among women who attended group antenatal care (G-ANC) in eight health centers in Machakos County, Kenya. Contrary to expectations, the majority of women delivered in facilities other than where they received G-ANC, with most choosing public hospitals equipped to provide comprehensive emergency obstetric and newborn care (CEmONC). This finding aligns with prior research in Kenya, which showed that some women frequently bypass nearby facilities, regardless of socioeconomic status due to concerns about quality of care [23]. Many prefer public hospitals that are perceived as better equipped and are willing to travel farther to access them [5]. These findings suggest that even when women engage with G-ANC, their delivery decisions are largely shaped by the perceived capacity of facilities to manage complications, rather than continuity with the G-ANC site.

At the same time, our findings reveal that proximity to the facility remained a key influence for a smaller group of women who delivered at the same G-ANC site. This highlights an important nuance: while some women are willing and able to travel farther for perceived higher-quality care, others, particularly those facing geographic or financial constraints tend to prioritize proximity. In our study, women in the higher wealth quintiles (4 and 5) were more likely to deliver at a different, typically better-equipped facility, suggesting that wealth enabled greater mobility and choice. This duality reinforces earlier research showing that proximity and perceived quality interact in complex ways to shape care-seeking behavior [4, 6, 12].

We also observed that staff attitudes were a strong predictor of whether women returned to their G-ANC facility for delivery, with a fivefold increased likelihood among those who reported positive staff attitudes. This suggests that G-ANC may influence delivery decisions not only through the content or structure of care, but also through the quality of interpersonal relationships formed during antenatal visits. Notably, a significantly higher proportion of women who delivered at the same facility where they attended G-ANC reported that the G-ANC experience influenced their delivery site choice. However, in adjusted analyses (Table 3), overall satisfaction with the G-ANC experience was not significantly associated with facility choice, raising the possibility that what women valued most in their G-ANC experience were the respectful and supportive staff attitudes. This underscores the importance of client-provider relationships in shaping care-seeking behaviors. This finding aligns with the G-ANC theory of change [7], which posits that strengthened interpersonal connections, and trust built during group care may influence subsequent service utilization, including delivery decisions. While we cannot conclusively attribute improved staff attitudes to the G-ANC intervention itself, our findings reinforce a growing body of evidence emphasizing the role of respectful maternity care in promoting continuity of care [2, 19, 21] and fostering trust in healthcare systems [2, 21, 25].

Advice received during pregnancy/ANC was significantly associated with women delivering at a different facility than where they received G-ANC. While our study did not ask where women sought such advice, evidence from other settings suggests that pregnant women receive guidance from a wide spectrum of sources, including healthcare professionals (midwives, obstetricians, and general practitioners), traditional birth attendants (TBAs), peers, family members, and increasingly, digital platforms such as social media, forums, and online communities [1, 3, 22]. For some women, professional guidance is highly valued and central to decision-making, while for others, advice is filtered through personal experience, cultural norms, or collective wisdom shared in their social networks [22]. In Ethiopia, for instance, [1], found that midwives often played a pivotal role in encouraging facility-based delivery by sympathetically advising women to seek care at referral hospitals when complications were anticipated. Women in that study also emphasized the importance of respectful communication, valuing providers who were polite, patient, reassuring, and consistently accessible. Similarly, in Kenya, Traditional Birth Attendants (TBAs) were described as integral members of the community, with their familiarity and availability making them key sources of advice and support throughout pregnancy [3]. Taken together, these findings suggest that ANC advice whether formal or informal functions not only as a mechanism for risk-based referrals but also as a form of social influence. In our study, it is likely that professional recommendations to seek delivery in better-equipped facilities contributed to the high proportion of women opting to deliver in CEmONC hospitals. However, future research should explore the dynamics of advice-seeking in G-ANC settings more directly, particularly in the context of evolving digital information sources.

Household wealth status was significantly associated with delivery facility choice in our study. Specifically, women from households in the two highest wealth quintiles (quintiles 4 and 5) were more likely to deliver in a different facility than the one where they received G-ANC. This finding suggests that wealthier women may have greater autonomy and resources to seek care at facilities they perceive to be of higher quality or better equipped most notably, Comprehensive Emergency Obstetric and Newborn Care hospitals, where the majority of these women delivered. Our findings mirror broader trends in sub-Saharan Africa, where quality maternal health service utilization remains highly inequitable [10, 23]. In Kenya, both the coverage and quality of maternal care have been shown to improve with increasing household wealth. Women in the lowest wealth quintile are significantly less likely to receive even the most basic, evidence-based interventions during childbirth compared to their wealthier counterparts [23]. Similarly, in Ethiopia, maternal health service utilization is disproportionately concentrated among wealthier women, with socio-economic barriers limiting access to quality care for those in lower-income groups [18]. These patterns underscore the persistent structural and financial inequalities that shape maternal health service utilization in Kenya, even when services are nominally free. Quality indicators increased with wealth, reinforcing how poverty not only limits access but also compromises the standard of care received. Taken together, these findings underscore that while financial protection schemes such as Linda Mama (a Kenyan government program providing free maternity services to pregnant women, mothers, and newborns) [30] are essential, they may be insufficient on their own to address entrenched socio-economic inequities. Women from wealthier households likely have greater mobility, access to information, and ability to act on provider advice to seek care in higher-quality or referral facilities. This suggests that policies aiming to improve maternal health outcomes must go beyond fee removal and prioritize both quality improvement and targeted support for the poorest and most marginalized women.

Relatedly, an interesting observation from our study is that affordability or cost-related barriers were not significantly associated with women’s choice of delivery facility. This may be partly explained by the widespread availability of free maternity services in Kenya’s public sector through the Linda Mama program. However, our study did not collect individual-level data on enrollment, awareness, or perceived entitlements under Linda Mama, which limits our ability to fully understand the role of financial protection in shaping delivery decisions. Although healthcare providers involved in the parent implementation study reported enrolling women into Linda Mama during G-ANC sessions, we were unable to verify whether women knew of their coverage or factored it into their choice of facility. As such, while user fees may no longer be a primary barrier, underlying disparities in service quality, non-financial costs like facility infrastructure and perceived competence of healthcare providers may still differentially influence facility choice across socio-economic groups [20].

Study limitations include: the phone administered questionnaire excluded women without mobile phone access possibly skewing the study population towards wealthier and less rural/remote participants. Also, data were collected for women who gave birth between August and December so seasonality of factors is not reflected (e.g., during the rainy season, October to December access could influence choice of facility). Machakos, as a comparatively peri-urban/urban county where women may have many more facility options to choose from given its proximity to Nairobi, may have attenuated our effects; replicating this study in rural/remote counties offering G-ANC is recommended. Additionally, the sample size estimation was based on provider-reported estimates of delivery patterns rather than population-level data, which may introduce bias; causal inferences cannot be made, and findings should be interpreted as indicative rather than definitive. The study facilities excluded hospitals providing G-ANC, which limited our ability to directly assess key factors such as complications requiring advanced care or the choice to deliver at public CEmONC facilities. Further, while our GEE model adjusted for facility caseload, more complex analyses, such as assessing the mediation and/or moderation effects of G-ANC with other factors were beyond the scope of this study. Future research should explore these relationships to deepen understanding of the mechanisms driving the observed outcomes.

Further, future research for sub-Saharan African settings with high rates of facility deliveries should examine factors influencing delivery facility choice including where G-ANC is being provided, while settings with low rates should explore if G-ANC has a positive effect on increasing facility delivery. Other research questions of interest include: how the household wealth status explicitly affects facility choice particularly among the poorest; how women understand their choices when enrolled in health insurance programs, such as Linda Mama that cover delivery costs; and bypassing and self-referral behaviors for maternity care within insurance schemes.

## Conclusions

The facility delivery decision is shaped by factors other than group antenatal care. G-ANC participants in health centers predominantly chose to deliver in another facility (i.e., not the same G-ANC facility). Most women gave birth in a public facility, and over half chose a CEmONC site. The choice of a different facility is shaped by health advice given during ANC/pregnancy and high household wealth status. The choice to give birth in the same facility where they received G-ANC is influenced by proximity and positive staff attitudes. These findings can guide future county and national programming related to G-ANC and other health system strategies aiming to improve maternal health outcomes.

## Supplementary Information

Additional file 1.

## Data Availability

De-identified datasets analysed during the current study are available on the https://figshare.com/account/items/28070636/edit

## Abbreviations

ANC: Antenatal care
BEmONC: Basic emergency obstetric and newborn care
CEmONC: Comprehensive emergency obstetric and newborn care
G-ANC: Group antenatal care
GEE: Generalized estimating equations
ICC: Inter-class coefficient
ICRHK: International centre for reproductive health - kenya
LMICs: Low- and middle-income countries
SDR: Service delivery redesign
UHC: Universal health care
