# Supplementary material for: Determinants of health facility choice for delivery among women participating in group antenatal care in Machakos county, kenya: A cross-sectional survey
Source: Reprod Health. 2025 Aug 22;22:150. doi: 10.1186/s12978-025-02107-w (PMC12374317; doi:10.1186/s12978-025-02107-w)
Supplement: Supplementary file 1 — Additional file 1. [file 12978_2025_2107_MOESM1_ESM.docx]

**Supplemental Table 1: Distribution of all factors (multiple responses) reported by participants influencing their choice of delivery facility**

| **Factor/variable at most recent pregnancy** | | **Delivered the same facility**  **(n =140)**  ***Number (%)*** | **Delivered at a different facility**  **(n=330)**  ***Number (%)*** |
| --- | --- | --- | --- |
| Health | Had complications during pregnancy | 3 (2.1) | 55 (16.7)*** |
|  | Complications during labor | 3 (2.1) | 45 (13.6)*** |
|  | Previous poor outcomes/ experiences with childbirth | 4 (2.9) | 18 (5.5) |
|  | Was told/advised to give birth there during pregnancy/ANC | 10 (7.1) | 57 (17.3)** |
|  | Was referred here when I was in labor | 5 (3.6) | 64 (19.4%)*** |
|  | Safety | 10 (7.1) | 36 (10.9) |
|  | Complication prevention | 5 (3.6) | 66 (20.0)*** |
|  | Sudden onset of labour | 4 (2.9) | 21 (6.4) |
|  | First pregnancy | 6 (4.3) | 49 (14.9)** |
| Access | A place I could afford to give birth (cost) | 20 (14.3) | 21 (6.4)** |
|  | Based on available transport | 21 (15.0) | 29 (8.8)* |
|  | A place I could safely get to when I was in labor (security) | 20 (14.3) | 33 (10.0) |
|  | Closest facility to my residence | 62 (44.3) | 51 (15.6)*** |
|  | Closest facility to my location when I went into labor | 12 (8.6) | 27 (8.2) |
| Facility | Had 24/7 services | 0 (0) | 23 (7.0)** |
|  | Staff attitudes | 37 (26.4) | 20 (6.1)*** |
|  | Trust | 21 (15.0) | 18 (5.5)*** |
|  | Good experience here (treated respectfully) | 43 (30.7) | 32 (9.7)*** |
|  | Able to have a birth companion | 4 (2.8) | 1 (0.3)* |
|  | Better package of care in a private facility | 0 (0) | 10 (3.0)* |
|  | Already enrolled in Linda Mama (have the card, so free of charge) | 10 (7.1) | 15 (4.6) |
|  | Enough staff working there | 3 (2.1) | 10 (3.0) |
|  | Availability of skilled providers (doctor, midwives) | 7 (5.0) | 20 (6.1) |
|  | Not too busy (caseload) | 3 (2.1) | 6 (1.8) |
|  | Other facilities have stockouts/supplies issues | 1 (0.7) | 6 (1.8) |
|  | CEmONC emergency services including C-section | 6 (4.3) | 70 (21.2)*** |
|  | Good quality of ANC/care | 26 (18.6) | 6 (1.8)*** |
|  | Poor experience/quality | 0 (0.0) | 5 (1.5) |
|  | Referred | 2 (1.4) | 28 (8.50)** |
|  | Can manage complications | 0 (0.0) | 28 (8.5)*** |
| Other (social) | Friends/peers influenced decision | 5 (3.6) | 19 (5.8) |
|  | Husband/family influenced decision (advice) | 0 (0.0) | 18 (5.5)* |
|  | Personal preference | 10 (7.1) | 50 (15.2)* |
| Birth preparation before the baby was born | Identified a facility where they planned to give birth | 136 (97.1) | 309 (93.6) |
|  | Planned how they were going to get there | 137 (98.0) | 313 (94.8) |
|  | Planned who was going to go with and help them | 136 (97.1) | 326 (98.8) |
|  | Saved money in case of an emergency | 139 (99.3) | 316 (95.8)* |
|  | Decided who could make decisions in the case of emergency | 135 (96.4) | 315 (95.5) |
|  | Prepared a birth kit | 140 (100) | 323 (97.9) |
| G-ANC Intervention | Liked G-ANC experience at this facility | 63 (45.0) | 38 (11.5)*** |
|  | Comfortable with the providers I know from G-ANC at this facility | 66 (47.1) | 2 (0.6)*** |
|  | Learned about birth planning during G-ANC | 24 (17.1) | 3 (0.9)*** |
|  | Selected this facility during birth planning | 36 (25.7) | 39 (11.8) |
| Key: *p < 0.05; ** p < 0.01; *** p < 0.001. | | | |
